# PReS-FINAL-1020: Dysregulation of the peripheral blood D cell compartment is associated with disease activity in juvenile dermatomyositis

**DOI:** 10.1186/1546-0096-11-S2-O3

**Published:** 2013-12-05

**Authors:** C Piper, H Varsani, K Arnold, L Wedderburn, C Mauri, K Nistala

**Affiliations:** 1 Centre for Rheumatology, Division of Medicine, University College London, UK; 2 Rheumatology Unit, UCL Institute of Child Health, London, UK

## Introduction

Juvenile dermatomyositis (JDM) is an autoimmune disease characterized by proximal muscle weakness and cutaneous manifestations, typically heliotrope rash and Gottron's papules. Previous studies have identified an increase in circulating B cells in JDM patients, but their provenance and functional characteristics have not been examined. In this study we investigated whether a recently identified immature B cell subset (CD24^hi^CD38^hi^) with known regulatory function (Breg) was enriched in JDM and correlated with disease outcome measures.

## Objectives

To characterize the peripheral blood B cell compartment in JDM patients, both before and after treatment, and assess the production of the B cell immunoregulatory cytokine, IL-10, in 45 patients recruited through the UK JDM Cohort and Biomarker Study.

## Methods

B cell subpopulations from peripheral blood mononuclear cells (PBMC) isolated from healthy controls (HC) and JDM patients were analyzed by flow cytometry using the surface markers CD19, CD24, CD38, CD27, IgM, and IgD. PBMC were stimulated for 72 hours with CD40 ligand (CD40L) transfected CHO cells, together with PMA and ionomycin for the last 5 hours in the presence of Brefeldin A. Cells were then stained for CD19 and intra-cellular IL-10 which was detected by flow cytometry.

## Results

Ex-vivo analysis of pre-treatment (pre-Rx) JDM PBMC displayed a significant increase in CD19^+ ^B cells when compared to post-Rx JDM samples (mean of 25.8% to 16.1%, p = 0.012). The immature B cell compartment was expanded in pre-Rx JDM patients (mean of 28.3% vs 12.3% in post-Rx, p = 0.0019). Moreover, this B cell expansion correlated with the severity of clinical disease (Physician's global assessment vs B cell frequency, R = 0.38). Following stimulation with CD40L, a lower proportion of B cells from JDM patients expressed IL-10 than aged matched controls (mean of 16.3% vs 18.9% respectively). JDM patients receiving corticosteroid treatment showed a trend for a higher frequency of CD40L induced IL-10+ B cells (mean 19.5% vs 14.4%, p = 0.12).

## Conclusion

These data identify an expansion of immature B cells in JDM patients at disease presentation. Although phenotypically similar to Breg, B cells from JDM patients did not match the induction of IL-10 seen in child controls. Ongoing work will test the hypothesis that Breg are defective in JDM, but regain IL-10 expression following immunomodulatory therapy.

## Disclosure of interest

None declared.

